# Identification and target prediction of miRNAs specifically expressed in rat neural tissue

**DOI:** 10.1186/1471-2164-10-214

**Published:** 2009-05-09

**Authors:** You-Jia Hua, Zhong-Yi Tang, Kang Tu, Li Zhu, Yi-Xue Li, Lu Xie, Hua-Sheng Xiao

**Affiliations:** 1 Bioinformatics Center, The Center of Functional Genomics, Key Lab of Systems Biology, Shanghai Institutes for Biological Sciences, Chinese Academy of Sciences, 320 Yueyang Road, Shanghai 200031, PR China; 2 Department of Gene Chip, National Engineering Center for Biochip at Shanghai, 151 Libing Road, Shanghai 201203, PR China; 3 Department of Translational Medicine, Shanghai Center for Bioinformatics Technology, 100 Qinzhou Road, Shanghai 200235, PR China; 4 Graduate School of the Chinese Academy of Sciences, 320 Yueyang Road, Shanghai 200031, PR China

## Abstract

**Background:**

MicroRNAs (miRNAs) are a large group of RNAs that play important roles in regulating gene expression and protein translation. Several studies have indicated that some miRNAs are specifically expressed in human, mouse and zebrafish tissues. For example, miR-1 and miR-133 are specifically expressed in muscles. Tissue-specific miRNAs may have particular functions. Although previous studies have reported the presence of human, mouse and zebrafish tissue-specific miRNAs, there have been no detailed reports of rat tissue-specific miRNAs. In this study, Home-made rat miRNA microarrays which established in our previous study were used to investigate rat neural tissue-specific miRNAs, and mapped their target genes in rat tissues. This study will provide information for the functional analysis of these miRNAs.

**Results:**

In order to obtain as complete a picture of specific miRNA expression in rat neural tissues as possible, customized miRNA microarrays with 152 selected miRNAs from miRBase were used to detect miRNA expression in 14 rat tissues. After a general clustering analysis, 14 rat tissues could be clearly classified into neural and non-neural tissues based on the obtained expression profiles with p values < 0.05. The results indicated that the miRNA profiles were different in neural and non-neural tissues. In total, we found 30 miRNAs that were specifically expressed in neural tissues. For example, miR-199a was specifically expressed in neural tissues. Of these, the expression patterns of four miRNAs were comparable with those of Landgraf et al., Bak et al., and Kapsimani et al. Thirty neural tissue-specific miRNAs were chosen to predict target genes. A total of 1,475 target mRNA were predicted based on the intersection of three public databases, and target mRNA's pathway, function, and regulatory network analysis were performed. We focused on target enrichments of the dorsal root ganglion (DRG) and olfactory bulb. There were four Gene Ontology (GO) functions and five KEGG pathways significantly enriched in DRG. Only one GO function was significantly enriched in the olfactory bulb. These targets are all predictions and have not been experimentally validated.

**Conclusion:**

Our work provides a global view of rat neural tissue-specific miRNA profiles and a target map of miRNAs, which is expected to contribute to future investigations of miRNA regulatory mechanisms in neural systems.

## Background

MiRNAs are a large class of tiny non-coding RNAs (~22 nt long). They have been identified in many species and their sequences have been published in databases [1]. MiRNAs regulate a large number of genes in animals and plants by binding to the 3'UTR or other regions of target mRNAs leading to degradation or translational repression during development, cell lineage division, and tumor generation [2-5]. In animals, miRNA transfection experiments showed that target genes are regulated by repression. However, increased evidences demonstrated that even in animals, target mRNAs can be degraded by miRNAs that also play key roles in the processes of tumorigenesis and cancer development [6,7].

MiRNA microarray technology is an efficient method to generate miRNA expression profiles. These microarray data can be used to extract information regarding the regulatory pathways initiated by miRNAs, especially regulation due to degradation, by integrating the mRNA expression profiles of predicted miRNA target genes. Such an approach has been applied to study the functional linkage between miRNAs and physiological or pathological processes [8-10].

Recently, Thomson and his colleagues [11] used miRNA microarray technology to study miRNA expression in mice. They demonstrated that there is a relationship between the expression profiles and the staged embryo temporal regulation of a large class of miRNAs, such as members of the *let-7 *family. Wienholds et al. [12], using microarrays with locked-nucleic acid-modified oligonucleotide probes, determined the temporal and spatial expression patterns of 115 conserved vertebrate miRNAs in zebrafish embryos. They found that most of the miRNAs were expressed in a highly tissue-specific manner during different developmental stages and physiological processes.

Several studies have indicated that some miRNAs are specifically expressed in human, mouse and zebrafish tissues [4,12-20]. Because the rat is a general animal model for biological research, tissue-specific expression of miRNAs has recently been studied in this model. Wang et al. [21] investigated the tissue-specific expression of miRNAs in six rat tissues (lung, heart, brain, kidney, liver and spleen), and found that miR-195 and miR-200c were expressed specifically in the lung. Their work suggested that there is some functional relevance between the lung-specific miRNAs identified and the normal physiological and pathological processes of the lung. Landgraf et al. [18] sequenced over 250 small RNA libraries from 26 tissue systems and cell types in human, mouse, and rat, providing a mammalian miRNA expression atlas. To study miRNA expression in the rat, they used six neural tissues or cell types (cortex, hippocampus, striatum, glioma, neuroblastoma and pheochromocytoma) and one non-neural tissue (thyroid) to generate miRNA expression profiles. The expression of miRNAs in the vertebrate central nervous system, such as human, mouse and zebrafish, has been previously reported [4,14-19]. For example, Bak et al. reported 13 mouse central nervous system enriched miRNA and found that 44 miRNAs were more than threefolds enrichment in the brain regions. Kapsimali et al. studied 38 abundant conserved miRNAs in developing and adult zebrafish brain by using *in situ *hybridization method and indicated that miRNAs have a different profile in neural cells.

Previous studies mainly focus on miRNA expression in human, mouse and zebrafish central nervous system or brain different regions. In order to investigate the differences in miRNA expression profiles in rat neural and non-neural tissues in detail, unlike the previous reports, we deliberately selected seven rat neural tissues, including central and peripheral neural tissues (olfactory bulb, cortex, hippocampus, brain stem, hypothalamus, spinal cord and dorsal root ganglion) and seven non-neural tissues (heart, lung, muscle, spleen, testicle, kidney and liver). Of these tissues, only two neural tissues (cortex and hippocampus) were previously studied by Landgraf et al. [18] to analyze the miRNA expression profiles by sequencing.

Furthermore we investigated the functional information of these neural tissue-specific miRNAs and the related regulatory networks, and the target genes of the selected miRNAs predicted by three public datasets (TargetScan, PicTar and miRanda) were studied in detail by function and pathway enrichment analysis. Thus, a global view of rat neural-specific miRNA expression profiles and their target maps was developed in this study.

## Results

### Main procedure

**Figure 1:**
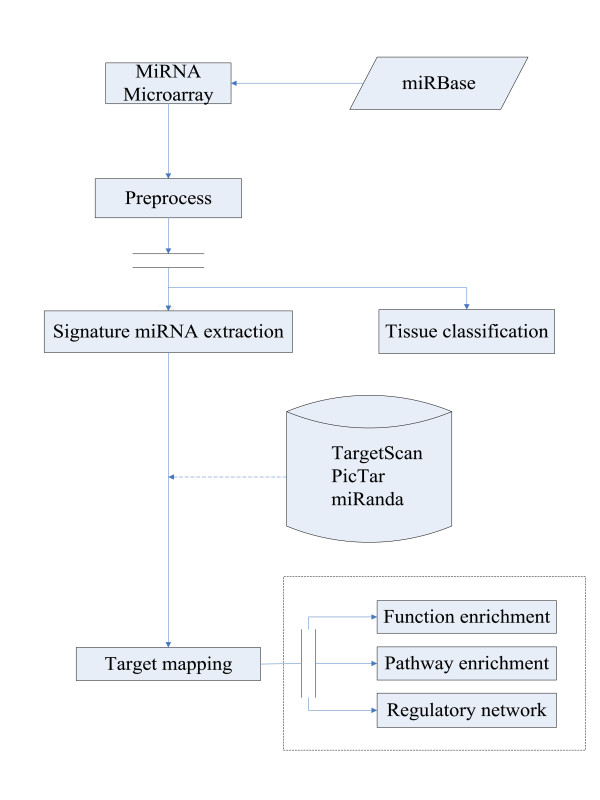
**The overall procedure**. The overall procedure of neural tissue-specific miRNA identification and functional analysis of targets was composed of four parts: obtaining miRNA expression profiles of 14 rat tissues, classification of tissues, extraction of signature miRNAs, and target mapping.

In this study, experiments and bioinformatics methods were integrated to create a global view of rat neural tissue-specific miRNAs, thus allowing their targets and functions to be identified (Fig. 1).

### MiRNA expression profiles of 14 rat tissues

In this study, miRNA tissue expression patterns were studied in seven rat neural tissues (olfactory bulb, cortex, hippocampus, brain stem, hypothalamus, spinal cord, and dorsal root ganglion) and seven non-neural tissues (heart, lung, muscle, spleen, testicle, kidney, and liver). In our previous study, we established an oligonucleotide microarray platform to detect miRNA signals [22]. A total of 192 different probes including 20 rat precursor miRNAs and 20 internal controls were designed for the production of customized miRNA microarrays, which represented 152 rat miRNAs. Tissue-specific miRNA expression profiling was performed based upon this platform. The correlation coefficients of repeated experiments were greater than 0.9 in the microarray experiments. The present call rate among all the microarrays was from 36% to 74%, with the average present rate being 56%. The print-tip loess normalization method was chosen for pre-processing the raw data because this method yields high quality normalized microarray data [23]. An expression data matrix of 152 miRNAs was produced, and this excluded all of the precursors and controls.

**Figure 2:**
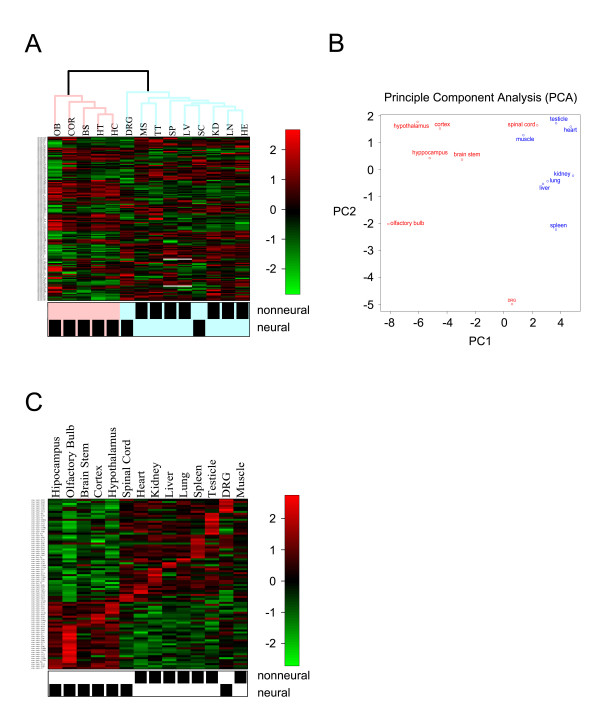
**MiRNA expression profiles**. The expression profiles of neural and non-neural tissues were shown by a) hierarchical clustering methods and b) principal component analysis (PCA) methods. BS: brain stem; COR: cortex; DRG: dorsal root ganglion; H: heart; HC: hippocampus; HT: hypothalamus; KD: kidney; LV: liver; LN: lung; MS: muscle; OB: olfactory bulb; SC: spinal cord; SP: spleen; TT: testicle. The highly-expressed miRNAs in each tissue were rearranged by a heat map in c). The bars in a) and c) denote the ratio of relative miRNA expression levels divided by the spike in oligo signals.

Fourteen tissues were clustered using a hierarchical clustering strategy [24] based upon the expression data matrix of the miRNAs that was produced. From the clustering results, the neural and non-neural tissues were correctly grouped into two classes (12 of 14, Fisher's test: p < 0.05) (Fig. 2a), except for two tissues, the dorsal root ganglion (DRG) and spinal cord. These tissues are generally considered part of the peripheral nerve system and were not grouped into the central neural tissue class (Fig. 2a), and this may be due to the peripheral location or intrinsic physiological properties of these two tissues.

For further confirmation and visualization of the above classifications, principal component analysis (PCA) was also performed, and similar results were obtained (Fig. 2b).

### Identification of neural tissue-specific miRNAs

**Figure 3:**
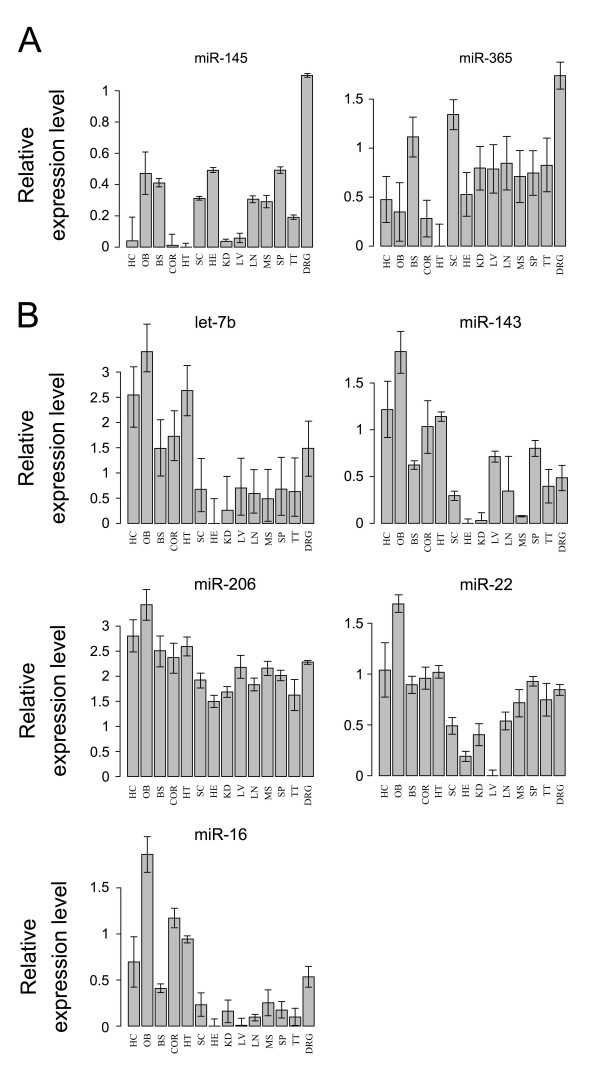
**Neural tissue-specific miRNAs in the DRG and olfactory bulb**. Neural tissue-specific miRNAs in a) DRG and b) olfactory bulb. The Y-axis denotes log_2 _value of the relative expression levels. BS: brain stem; COR: cortex; DRG: dorsal root ganglion; H: heart; HC: hippocampus; HT: hypothalamus; KD: kidney; LV: liver; LN: lung; MS: muscle; OB: olfactory bulb; SC: spinal cord; SP: spleen; TT: testicle.

Signature miRNAs for each tissue were extracted from neural tissues for comparison of neural miRNA expression profiles with non-neural tissues using the moderated t-test and empirical Bayesian methods. In total, 30 relatively specifically expressed miRNAs in neural tissues were found with p < 0.1 (see Table 1 and Additional file 1). These miRNAs have relatively higher expression levels in neural tissues than in the seven non-neural tissues. The olfactory bulb had the most neural-specific miRNAs; there were 26 miRNAs highly expressed in the olfactory bulb (Table 1). The DRG expressed eight miRNAs at high levels. Furthermore, there were seven miRNAs that were only expressed at high levels in one neural tissue, for example let-7b, miR-16, miR-22, miR-206, and miR-143 specifically expressed in olfactory bulb (Fig. 3b). MiR-145 and miR-365 were specifically expressed in DRG (Fig. 3a). In order to intuitively detect tissue-specific miRNA signatures, the miRNA expression matrix was rearranged as in Figure 2c. Eight miRNAs were randomly selected and validated by real-time PCR, and the data were reported in our previous study [23].

**Table 1:** Rat neural tissue specifically expressed miRNAs

Tissue name	MiRNA name
Hippocampus	let-7c, let-7c-2, miR-128a, miR-124a-1, miR-124a-3, miR-148b, miR-150, miR-199a, miR-217, miR-28, miR-29b-1, miR-329, miR-331.
Olfactory bulb	let-7b, let-7c-1, let-7c-2, miR-10a, miR-16, miR-17, miR-21, miR-22, miR-28, miR-29c, miR-124a-1, miR-124a-3, miR-128a, miR-135b, miR-143, miR-148b, miR-150, miR-199a, miR-206, miR-217, miR-223, miR-29b-1, miR-329, miR-331, miR-429, miR-451.
Cortex	let-7c-1, miR-10a, miR-21, miR-124a-1, miR-128a, miR-135b, miR-150, miR-199a, miR-217, miR-329, miR-451.
Hypothalamus	miR-17, miR-29c, miR-124a-1, miR-128a, miR-150, miR-199a, miR-217, miR-223, miR-329, miR-429.
Spinal cord	miR-28, miR-217, miR-218-1, miR-329, miR-331.
Brain stem	let-7c-1, miR-17, miR-135b, miR-150, miR-199a, miR-218-1, miR-223, miR-329.
Dorsal root ganglion	let-7c, miR-17, miR-145, miR-150, miR-199a, miR-223, miR-365, miR-451.

### Target mapping and functional annotation

To further investigate the functional significance of these neural tissue-specific miRNAs and related regulatory networks, heuristic information of the miRNA target genes was needed. By integrating three public databases (TargetScan, PicTar and miRanda), 1,475 target genes of the 30 neural tissue-specific miRNAs were collected (see Additional file 2). Comparing with targets of randomly selected miRNAs by hypergeometric test (p < 0.05), the results indicated that these 1,475 targets were not obtained by chance (see Additional file 3). Of the seven neural tissues, the specific miRNAs in the olfactory bulb had 1,442 target genes, which was a much greater number of targets than the other neural tissues. These 1,475 target genes were then carefully annotated by the GO function, KEGG pathway, and regulatory network enrichment analysis.

**Figure 4:**
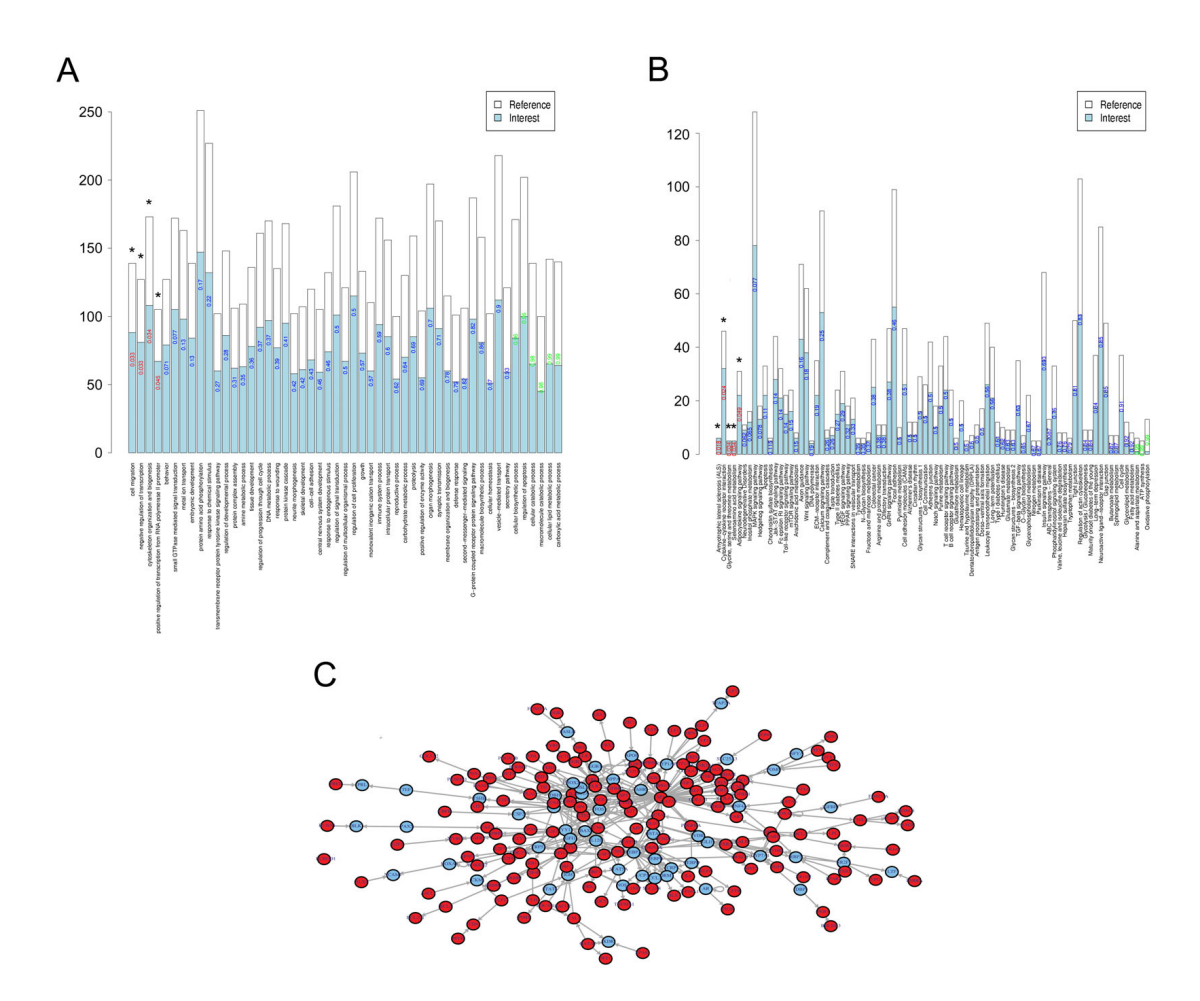
**Target mapping of neural tissue-specific miRNAs in the DRG**. Targets of neural tissue-specific miRNAs mapping in a a) function enrichment graph, b) pathway enrichment graph and c) regulatory network graph (by the Steiner tree algorithm) in the DRG. In a), each bar represented the targets of both highly-expressed and less-expressed miRNAs, which were annotated to the GO database in the DRG. The blue column represented the total number of these targets. The number in each bar represents the p value obtained using Fisher's test. The significant p values are in red (p < 0.05) and non-significant p values are in green (p > 0.95). In b), the red parts represent the targets of specifically expressed miRNAs in the heat map. In c), genes in red represent targets of tissue-specific miRNAs, the blue shows genes that are not targets of tissue-specific miRNAs, and the arrows represent the direction of regulation.

**Figure 5:**
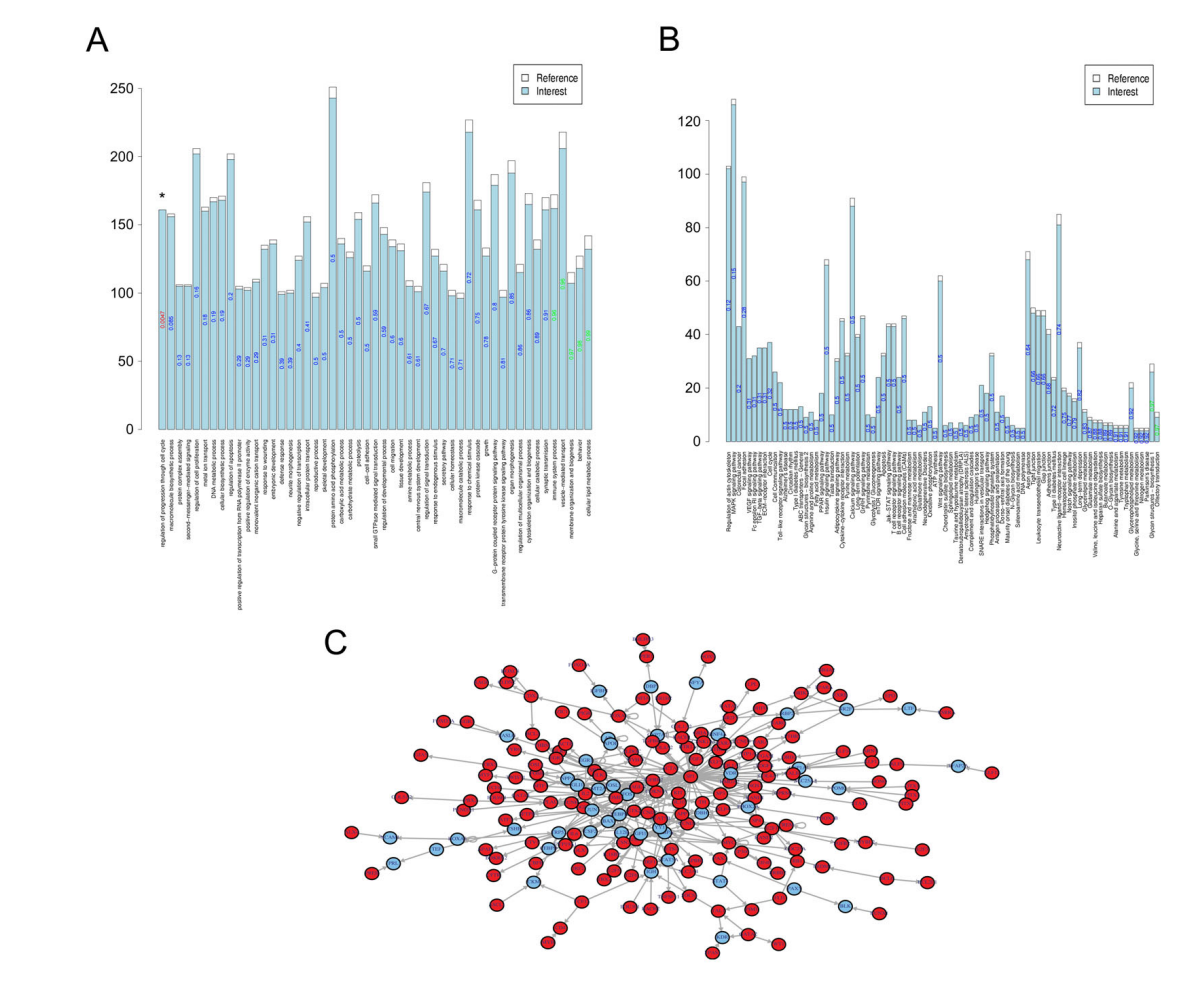
**Target mapping of neural tissue-specific miRNAs in the olfactory bulb**. Targets of miRNA mapping in a a) function enrichment graph, b) pathway enrichment graph and c) regulatory network graph (by the Steiner tree algorithm) in the olfactory bulb. In a), each bar represented targets of both highly-expressed and less-expressed miRNAs, which were annotated to the GO database in the olfactory bulb. The blue column represents the total number of these targets. The number in each bar represents the p value obtained using Fisher's test. The significant p values are in red (p < 0.05) and non-significant p values are in green (p > 0.95). In b), the red parts represent targets of specifically expressed miRNAs in the heat map. In c), red represents genes that were targets of tissue-specific miRNAs, blue represents genes that were not targets of tissue-specific miRNAs, and the arrows represent the direction of regulation.

GO function and KEGG pathway enrichments were performed by mapping the predicted target genes from the neural tissue-specific miRNAs to the GO and KEGG databases, respectively. For these miRNAs, 51 GO functions and 84 KEGG pathways were annotated. Each tissue had its own enriched pathway and function (data not shown). In this study, we have mainly presented the GO function and pathway results from two tissues (olfactory bulb and DRG) (Fig. 4 &5). In the olfactory bulb, which had the highest number of neural tissue-specific miRNAs, "Regulation of progression through cell cycle" was the most enriched GO function (p = 0.005) (Fig. 5a). In the DRG, four GO functions (cell migration, p = 0.033; negative regulation of transcription, p = 0.033; cytoskeleton organization and biogenesis, p = 0.034; positive regulation of transcription from the RNA polymerase II promoter, p = 0.045) and five KEGG pathways (amyotrophic lateral sclerosis, ALS, p = 0.018; cytokine-cytokine receptor interaction, p = 0.024; glycine, serine and threonine metabolism, p = 0.034; seleno-amino acid metabolism, p = 0.034; adipocytokine signaling pathway, p = 0.049) were significantly enriched by the targets of 7 DRG specific miRNAs (Fig. 4a, b). On the other hand, we randomly selected 7 DRG non-specific miRNAs for 100 times and predicted their targets. Then, these targets were annotated to those functions or pathways enriched in DRG. In these functions and pathways, most of the average 100 p values were more than 0.05 (see Additional file 4). These results indicated that the enriched functions and pathways by the targets of 7 DRG specific miRNAs were significantly different from those obtained by chance.

Furthermore, in order to identify regulatory relationships amongst the target genes, we developed a strategy to construct general gene regulatory networks by Transfac v9.4 and to match target genes into the resulting networks using the Steiner Tree Algorithm. Finally, a specialized connected regulatory network was extracted for the predicted target genes that were classified by tissue-specific miRNAs. In the DRG, 161 target genes of the specific miRNAs were included in the regulatory network (Fig. 4c, red circles). Of these, the *SP1 *gene occupied the central position in the network, and it regulated many more downstream genes than other regulatory genes in the DRG. In the olfactory bulb, 160 target genes were included (Fig. 5c, red circles).

Three kinds of enrichment maps for function, pathway, and regulatory networks were generated for each tissue. Figures 4 and 5 show examples of the three maps for the miRNA targets expressed in the DRG and olfactory bulb, which may provide insights into the combinatorial regulation mechanisms initiated by tissue-specifically expressed miRNAs.

## Discussion

In this study, high-throughput miRNA microarray technology with 152 miRNAs selected from miRBase was used to detect miRNA expression in 14 rat tissues. The seven neural tissues selected included olfactory bulb, cortex, hippocampus, brain stem, hypothalamus, spinal cord, and dorsal root ganglion. The tissues selected represented both the central and peripheral nervous systems. The neural miRNA expression profiles were compared with profiles from seven other tissues, including heart, lung, muscle, spleen, testicle, kidney, and liver in order to identify neural-specific miRNAs. Although several studies have previously reported the existence of neural-specific miRNAs, only the striatum, cortex, and hippocampus were selected for these previous analyses. For example, Landgraf et al. [18] studied the mammalian miRNA expression atlas by sequencing 250 small RNA libraries representing 26 different human and rodent organ systems and cell types, and Wang et al. [21] used home-made miRNA microarrays to identify the rat lung-specific miRNAs, miR-195 and miR-200c. The number of tissues in this work was up to date the largest rat nerve system related tissue set. The miRNA expression data were integrated with carefully screened functional data from public databases using bioinformatics and bio-statistics based strategies. Tissue-specific miRNA expression profiles were obtained from rat neural and non-neural tissues, and the neural tissue-specific expression profile related miRNAs and their significant target genes. This provided a global view of rat tissue-specific miRNA profiles and their target maps, thus contributing to a better understanding of the role of miRNAs in neural systems. In comparison to the previous studies that investigated neural-specific miRNA expression, we have obtained a more detailed picture of rat tissue miRNA expression profiles using a larger number of neural tissue types. Moreover, based upon our analyses of tissue-specific expression profiles, the resulting signature miRNAs may be used as biomarkers for the classification of tissues from different sources.

After performing the data analysis, we found a total of 30 miRNAs that were specifically expressed in neural tissues. In them, four miRNAs (miR-9, miR-124, miR-128a and miR-128b) were previously reported to be specifically expressed in the cortex and hippocampus in rat [18]. Comparing with other animals such as mouse or zebrafish, the expression of several miRNAs agrees with previous study. For example, in mouse [14], miR-10b is highly expressed in spinal cord; miR-124 is widely expressed in brain tissues; miR-200b, miR-128a, miR-128b, miR-429 are specifically expressed in olfactory bulb; miR-200a is highly expressed in olfactory bulb; miR-7b is highly expressed in hypothalamus. Moreover, miR-200b is enriched in zebrafish olfactory bulb; miR-124 and miR-9 expression are detected throughout adult brain [16]. But more neural specifically expressed miRNAs were found in rat tissues in this study. Although the data were generated from completely different platforms or methods and different miRNA probe sources were used in these studies, several miRNA expression patterns were consistent with previous results, in which the same types of tissues were used in different animal models. Collectively, the data provides convincing evidence that tissue-specific miRNA expression processes and related regulatory mechanisms are highly stable, and may also indicate that the regulatory mechanisms mediated by miRNAs are conserved, and may play a more important role in physiological or pathological processes that previously believed. We identified a number of miRNAs specifically expressed in neural tissue; however, the functions and cell type distributions of most of the miRNAs were not clear. These will be studied in detail in the future.

Furthermore, targets of the specifically expressed miRNAs were mapped to gene function databases, pathway databases, and regulatory networks. This approach clearly illustrated that specifically expressed miRNAs and their targets perform integrated regulatory functions. For example, the fact that a large number of miRNAs were very highly expressed in the olfactory bulb but not in other tissues suggests that more specific combinatory regulation mechanisms may be performed by these miRNAs and their targets. The only enriched GO function in the olfactory bulb is "regulation of cell cycle", which could be affected by processes that modulate the rate or extent of progression through the cell cycle. In the DRG, there are more enriched functions, such as cell migration, cytoskeleton organization, and biogenesis, negative regulation of transcription, and positive regulation of transcription from the RNA polymerase II promoter. The pathways enriched in the DRG are those associated with amyotrophic lateral sclerosis, cytokine-cytokine receptor interactions, glycine, serine and threonine metabolism, seleno-amino acid metabolism, and the adipocytokine signalling pathway. These results imply that as the primary afferent sensory neuron, the DRG takes on a very important role in sensory signal transduction, conduction, and transmission. This study described the neural specifically expressed miRNA target genes, gene ontology and network information. Biological validation experiments needed to be further studied.

In this study, tissue-specific expressed miRNAs were also classified into different groups based on the information regarding miRNA families found on the Sanger website. Groups containing more than one miRNA were selected to compute inter-family and intra-family correlation coefficients. The analyses indicated that there were no significant differences between the correlation coefficients of intra-family and inter-family miRNAs (data not shown). This is not a surprising result, given that compensatory mechanisms of miRNAs are known to exist.

In summary, this study attempted to create a holistic view of rat tissue-specific miRNA expression profiles and combinatorial regulation mechanisms using seven neural tissues and seven non-neural tissues. By integrating the data and information obtained from function enrichment, pathway enrichment, and regulatory networks of tissue-specific miRNA targets, we also obtained regulatory networks mediated by tissue-specific miRNAs in an intuitive manner. Our strategies for extracting significant tissue-specific miRNA expression profiles, approaches for public data integration and extensive curated data sources may serve as valuable tools for the further experimental investigation of regulatory mechanisms of miRNAs in neural systems.

## Conclusion

In this study, we selected seven neural tissues and seven non-neural tissues and analyzed their tissue-specific miRNA expression profiles. After performing general clustering procedures, the 14 rat tissues can be clearly classified into neural and non-neural tissues based on their miRNA expression profiles. Thirty miRNAs were found to be specifically expressed in neural tissues. The neural-specific miRNAs were chosen to predict target genes based on three public datasets, and then a pathway analysis of these target genes was performed. The data from this study will contribute to further investigations of miRNA regulatory mechanisms in neural systems, as well as to functional studies.

## Methods

### Tissue preparation and total RNA isolation

Ten Sprague-Dawley male rats (body weight, 200–250 g) were used to prepare samples from the 14 normal tissues used in these experiments. Seven neural tissues (olfactory bulb, cortex, hippocampus, brain stem, hypothalamus, spinal cord, and dorsal root ganglion) and seven non-neural tissues (heart, lung, muscle, spleen, testicle, kidney, and liver) from each rat were selected. The experiments were approved by the Committee of Use of Laboratory Animals and Common Facility, Institute of Neuroscience, Chinese Academy of Sciences. Total RNAs from all the samples were extracted using the mirVana™ miRNA Isolation Kit (Ambion). The quality of the RNA was assessed with the Bioanalyzer 2100 (Agilent). Total RNAs from the same tissues of different rats were combined in order to eliminate individual diversity.

### MiRNA microarray platform

An oligonucleotide microarray platform was established to detect miRNA signals. A total of 152 rat miRNA probes, which were 40 nt long, were designed for the miRNA sequences. These sequences corresponded to the rat miRNAs published in the miRNA Registry . Moreover, 14 control probes were also designed, including eight rat tRNA sequences for positive controls and six *Arabidopsis thaliana *miRNA sequences for negative controls. Six blank control probes were also designed. Each probe was repeated three times in each microarray. In this microarray platform, the sample miRNA was labelled with Cy5, while spike in oligos were labelled with Cy3 for data normalization. The subsequent details of microarray fabrication, sample labelling, and microarray hybridization can be found in our previous study [22].

### Data pre-processing and validation

For the first step of the pre-processing procedure, the Cy5 signal was divided by the Cy3 signal with the spike in oligo sequence for each spot in the microarrays.

The print-tip loess method was used to normalize the miRNA microarray data according to our previous work [23]. The normalized microarray data was validated by using real-time PCR method [23].

### Clustering and principal component analysis

The hierarchical clustering method [24] was used to classify different tissue patterns. Principal component analysis (PCA) [25] was used to produce a two-dimensional graph of the distances between different tissues.

### Tissue-specific miRNA identification

The relative expression levels of each miRNA in the 14 tissues were compared pairwise by the moderated t-test. If the 13 comparisons of one miRNA in any tissue with the other tissues all had statistical significance (p < 0.1), then that miRNA was considered as tissue-specific in that particular tissue. The p value was adjusted by using the empirical Bayesian approach [26]. The significance of the difference between neural tissues and non-neural tissues was measured by the Fisher's test.

### Target prediction

Three types of miRNA target prediction software, TargetScan , PicTar , miRanda , were used to predict the target genes of 30 neural tissue specifically expressed miRNAs. The intersection of these three datasets was used as the prediction results of the target genes of 30 miRNAs.

### Enrichment information

The GO [27] package in R  was used to annotate the functions of the miRNA targets. If any function was mapped by more than 100 targets of tissue-specific miRNAs, then it was selected. The KEGG [28] package in R was used to annotate the pathways of the miRNA targets. If any pathway was mapped by more than five targets of the tissue-specific miRNAs, then it was selected. A hypergeometric test was used to validate the significance (p < 0.05) of the annotated information.

Regulatory networks were obtained from Transfac v9.4 . The targets of specific miRNAs were mapped to the networks. The Steiner Tree Algorithm was then used to find the connected sub-networks containing these targets [29].

The miRNA family information on the Sanger website was used to analyze the family classifications [1].

## Authors' contributions

YJH prepared the RNA samples, established the miRNA microarray platform and helped draft the manuscript. ZYT and LZ performed the data analysis, performed the target mapping and drafted the manuscript. YJH, ZYT and KT conceived of the study and participated in its design. YXL, LX and HSX participated in its design and helped draft the manuscript. All authors read and approved the final manuscript.

## Supplementary Material

Additional file 1 **List of p values of neural tissue-specific miRNAs**. The average p values of 30 neural specific miRNAs in each specific-expressed tissue versus other tissues by Fisher's test. BS: brain stem; COR: cortex; DRG: dorsal root ganglion; HC: hippocampus; HT: hypothalamus; OB: olfactory bulb; SC: spinal cord. Click here for file

Additional file 2 **Target intersection of three databases**. The intersection of TargetScan, PicTar and miRanda predicted target genes was shown with regard to 30 neural tissue-specific miRNAs. The intersection (light green color) is defined as the overlapping part among all of the three databases. Click here for file

Additional file 3 **Histogram of p value distribution**. 30 non-neural specific miRNAs were randomly selected for 1000 times, their targets were obtained, and they were compared them with the targets of 30 neural specific miRNAs to obtain 1000 p values by hypergeometric test. The distribution of the 1000 p values was shown as a histogram. Click here for file

Additional file 4 **List of p values of DRG enriched functions or pathways by random targets**. The average p values were obtained by annotating the targets of randomly selected DRG non-specific miRNAs to the functions or pathways enriched by the targets of DRG specific miRNAs by hypergeometric test. P > 0.05 denotes that these functions or pathways were not enriched by chance. Click here for file

## References

[B1] Griffiths-Jones S (2004). The microRNA Registry. Nucleic Acids Res.

[B2] Biemar F, Zinzen R, Ronshaugen M, Sementchenko V, Manak JR, Levine MS (2005). Spatial regulation of microRNA gene expression in the Drosophila embryo. Proc Natl Acad Sci USA.

[B3] Mendell JT (2005). MicroRNAs: critical regulators of development, cellular physiology and malignancy. Cell Cycle.

[B4] Miska EA, Alvarez-Saavedra E, Townsend M, Yoshii A, Sestan N, Rakic P, Constantine-Paton M, Horvitz HR (2004). Microarray analysis of microRNA expression in the developing mammalian brain. Genome Biol.

[B5] Reinhart BJ, Weinstein EG, Rhoades MW, Bartel B, Bartel DP (2002). MicroRNAs in plants. Genes Dev.

[B6] Lim LP, Lau NC, Garrett-Engele P, Grimson A, Schelter JM, Castle J, Bartel DP, Linsley PS, Johnson JM (2005). Microarray analysis shows that some microRNAs downregulate large numbers of target mRNAs. Nature.

[B7] Wang X (2006). Systematic identification of microRNA functions by combining target prediction and expression profiling. Nucleic Acids Res.

[B8] Garzon R, Pichiorri F, Palumbo T, Visentini M, Aqeilan R, Cimmino A, Wang H, Sun H, Volinia S, Alder H (2007). MicroRNA gene expression during retinoic acid-induced differentiation of human acute promyelocytic leukemia. Oncogene.

[B9] Mineno J, Okamoto S, Ando T, Sato M, Chono H, Izu H, Takayama M, Asada K, Mirochnitchenko O, Inouye M (2006). The expression profile of microRNAs in mouse embryos. Nucleic Acids Res.

[B10] Xu S, Witmer PD, Lumayag S, Kovacs B, Valle D (2007). MicroRNA (miRNA) transcriptome of mouse retina and identification of a sensory organ-specific miRNA cluster. J Biol Chem.

[B11] Thomson JM, Parker J, Perou CM, Hammond SM (2004). A custom microarray platform for analysis of microRNA gene expression. Nat Methods.

[B12] Wienholds E, Kloosterman WP, Miska E, Alvarez-Saavedra E, Berezikov E, de Bruijn E, Horvitz HR, Kauppinen S, Plasterk RH (2005). MicroRNA expression in zebrafish embryonic development. Science.

[B13] Xu C, Lu Y, Pan Z, Chu W, Luo X, Lin H, Xiao J, Shan H, Wang Z, Yang B (2007). The muscle-specific microRNAs miR-1 and miR-133 produce opposing effects on apoptosis by targeting HSP60, HSP70 and caspase-9 in cardiomyocytes. J Cell Sci.

[B14] Bak M, Silahtaroglu A, Moller M, Christensen M, Rath MF, Skryabin B, Tommerup N, Kauppinen S (2008). MicroRNA expression in the adult mouse central nervous system. RNA.

[B15] Hohjoh H, Fukushima T (2007). Expression profile analysis of microRNA (miRNA) in mouse central nervous system using a new miRNA detection system that examines hybridization signals at every step of washing. Gene.

[B16] Kapsimali M, Kloosterman WP, de Bruijn E, Rosa F, Plasterk RH, Wilson SW (2007). MicroRNAs show a wide diversity of expression profiles in the developing and mature central nervous system. Genome Biol.

[B17] Krichevsky AM, King KS, Donahue CP, Khrapko K, Kosik KS (2003). A microRNA array reveals extensive regulation of microRNAs during brain development. RNA.

[B18] Landgraf P, Rusu M, Sheridan R, Sewer A, Iovino N, Aravin A, Pfeffer S, Rice A, Kamphorst AO, Landthaler M (2007). A mammalian microRNA expression atlas based on small RNA library sequencing. Cell.

[B19] Sempere LF, Freemantle S, Pitha-Rowe I, Moss E, Dmitrovsky E, Ambros V (2004). Expression profiling of mammalian microRNAs uncovers a subset of brain-expressed microRNAs with possible roles in murine and human neuronal differentiation. Genome Biol.

[B20] Chen JF, Mandel EM, Thomson JM, Wu Q, Callis TE, Hammond SM, Conlon FL, Wang DZ (2006). The role of microRNA-1 and microRNA-133 in skeletal muscle proliferation and differentiation. Nat Genet.

[B21] Wang Y, Weng T, Gou D, Chen Z, Chintagari NR, Liu L (2007). Identification of rat lung-specific microRNAs by micoRNA microarray: valuable discoveries for the facilitation of lung research. BMC Genomics.

[B22] Zhao JJ, Hua YJ, Sun DG, Meng XX, Xiao HS, Ma X (2006). Genome-wide microRNA profiling in human fetal nervous tissues by oligonucleotide microarray. Childs Nerv Syst.

[B23] Hua YJ, Tu K, Tang ZY, Li YX, Xiao HS (2008). Comparison of normalization methods with microRNA microarray. Genomics.

[B24] Eisen MB, Spellman PT, Brown PO, Botstein D (1998). Cluster analysis and display of genome-wide expression patterns. Proc Natl Acad Sci USA.

[B25] Raychaudhuri S, Stuart JM, Altman RB (2000). Principal components analysis to summarize microarray experiments: application to sporulation time series. Pac Symp Biocomput.

[B26] Smyth GK (2004). Linear models and empirical bayes methods for assessing differential expression in microarray experiments. Stat Appl Genet Mol Biol.

[B27] Ashburner M, Ball CA, Blake JA, Botstein D, Butler H, Cherry JM, Davis AP, Dolinski K, Dwight SS, Eppig JT (2000). Gene ontology: tool for the unification of biology. The Gene Ontology Consortium. Nat Genet.

[B28] Kanehisa M, Goto S, Hattori M, Aoki-Kinoshita KF, Itoh M, Kawashima S, Katayama T, Araki M, Hirakawa M (2006). From genomics to chemical genomics: new developments in KEGG. Nucleic Acids Res.

[B29] Klein P (1995). A nearly best-possible approximation algorithm for node-weighted Steiner trees. J Algorithm.

