# Supplementary figures and images for: Identification and target prediction of miRNAs specifically expressed in rat neural tissue
Source: BMC Genomics. 2009 May 9;10:214. doi: 10.1186/1471-2164-10-214 (PMC2688525; doi:10.1186/1471-2164-10-214)

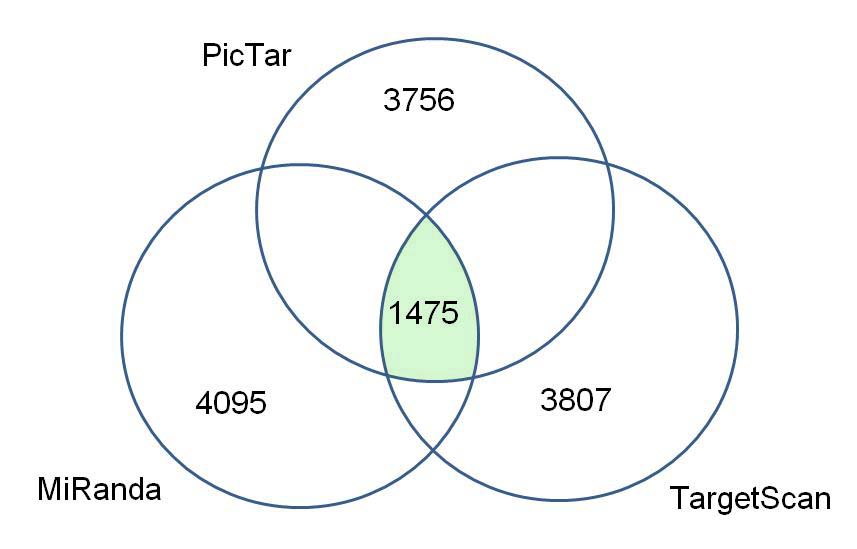

Supplement: Additional file 2 — Target intersection of three databases. The intersection of TargetScan, PicTar and miRanda predicted target genes was shown with regard to 30 neural tissue-specific miRNAs. The intersection (light green color) is defined as the overlapping part among all of the three databases. [file 1471-2164-10-214-S2.jpeg]

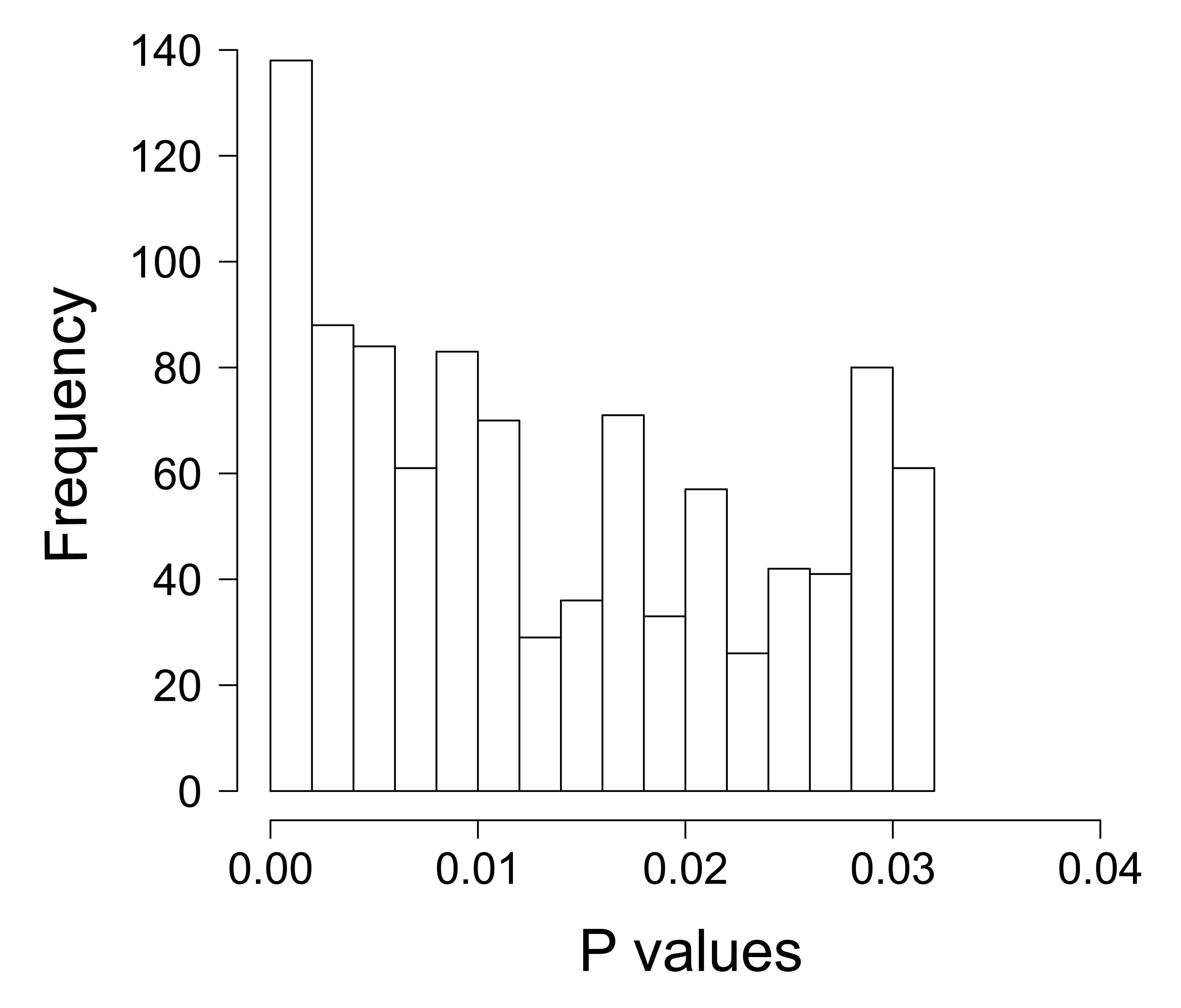

Supplement: Additional file 3 — Histogram of p value distribution. 30 non-neural specific miRNAs were randomly selected for 1000 times, their targets were obtained, and they were compared them with the targets of 30 neural specific miRNAs to obtain 1000 p values by hypergeometric test. The distribution of the 1000 p values was shown as a histogram. [file 1471-2164-10-214-S3.jpeg]
